# Andrographolide Prevents EV-D68 Replication by Inhibiting the Acidification of Virus-Containing Endocytic Vesicles

**DOI:** 10.3389/fmicb.2018.02407

**Published:** 2018-10-08

**Authors:** Dongyin Wang, Haoran Guo, Junliang Chang, Dong Wang, Bin Liu, Pujun Gao, Wei Wei

**Affiliations:** ^1^Department of Hepatology, The First Hospital of Jilin University, Jilin University, Changchun, China; ^2^Institute of Virology and AIDS Research, The First Hospital of Jilin University, Changchun, China; ^3^Changchun Institute of Biological Products, Changchun, China; ^4^Department of Hand Surgery, The First Hospital of Jilin University, Changchun, China

**Keywords:** enterovirus D68, andrographolide, endocytosis, acidification, drug development

## Abstract

Enterovirus D68 (EV-D68) has emerged as a significant respiratory pathogen that can cause severe respiratory disease and acute neurologic disease. At present, there are no approved antiviral agents or vaccines for EV-D68. In this study, we demonstrate that andrographolide (ADO), an active component of *Andrographis paniculata*, exerts substantial antiviral activity against EV-D68 infection. ADO treatment dramatically inhibited EV-D68 RNA replication (EC_50_ = 3.45 μM) and protein synthesis without producing significant cytotoxicity at virucidal concentrations. ADO-treated cells did not show any changes in host immune activation, EV-D68 attachment, or viral 5′ UTR activity. Using a pH-sensitive fluorescent indicator system for endocytosis in living cells, we found that ADO prevented the acidification of endocytic vesicles after receptor-mediated endocytosis. Finally, we showed that ADO inhibited the viral replication of circulating isolated EV-D68 strains. In summary, our results demonstrate that ADO suppresses EV-D68 replication by targeting the maturation of virus-containing endosomes of EV-D68. This mechanism represents a promising strategy for drug development.

## Introduction

The genus Enterovirus (EV) of the family Picornaviridae includes several medically important pathogens such as poliovirus, echoviruses, coxsackieviruses, numbered enteroviruses, and rhinoviruses (Baggen et al., 2018). In past decades, several enteroviruses have emerged as serious public health concerns. Recently, enterovirus D68 (EV-D68; originally classified as human rhinovirus 87) (Ishiko et al., 2002), caused a large outbreak of severe lower respiratory tract disease in North America. EV-D68 was first isolated in California from patients with pneumonia and bronchiolitis in 1962 (Schieble et al., 1967) and has since been infrequently reported in association with a wide range of clinical manifestations including mild-to-severe respiratory disease (Kaida et al., 2011; Huang et al., 2017). In 2014, the largest outbreak of EV-D68 infection occurred in the United States (Midgley et al., 2014, 2015). Most patients were children with severe respiratory conditions that necessitated intensive care. Patients with a history (Baggen et al., 2018) of asthma or reactive airway disease were more likely to require ventilator support (Midgley et al., 2015). Since the 2014 outbreak, an increasing number of clusters of EV-D68 infection have been reported in Europe, Asia, and Oceania (Messacar et al., 2015, 2016; Oermann et al., 2015; Reiche et al., 2015; Wei et al., 2016). EV-D68 has also been implicated in polio-like neurological disorders such as acute flaccid myelitis (Greninger et al., 2015; Maloney et al., 2015).

EV-D68 differs from other enteroviruses in that it is most often detected in cases of respiratory illness and has biological similarity to rhinovirus (Blomqvist et al., 2002; Oberste et al., 2004; Mertz et al., 2015). EV-D68 is a small, icosahedral virus with a single, positive-stranded RNA genome. The viral genome contains only 1 open reading frame (ORF) that encodes viral polyprotein, which is self-digested by viral proteins 2A and 3C to yield four structural proteins (VP1, VP2, VP3, and VP4) and seven non-structural proteins (2A, 2B, 2C, 3A, 3B, 3C, and 3D) (Baggen et al., 2018). The viral crystal structure of EV-D68 also shows similarity to that of human rhinovirus (Oberste et al., 2004). The EV-D68 capsid consists of 60 copies of each of the subunit proteins VP1, VP2, VP3, and VP4 and has a deep surface depression (canyon) circling each of the 12 pentameric vertices, which is thought to be responsible for viral receptor recognition (Liu et al., 2015b). Previous studies have suggested that cell surface sialic acid is required for host cell infection by EV-D68 (Liu et al., 2015a; Baggen et al., 2016). We recently identified neuro-specific intercellular adhesion molecule 5 (ICAM-5/telencephalin) as a functional entry receptor for sialic acid-dependent and -independent EV-D68 viruses (Wei et al., 2016).

Despite the spread of EV-D68 viruses, there are no effective vaccines or antiviral agents available for clinical use. It was recently reported that three antiviral drugs in clinical trials for enteroviral infections (pleconaril, pocapavir, and vapendavir) failed to inhibit EV-D68 infection (Liu et al., 2015b; Rhoden et al., 2015; Sun et al., 2015).

*Andrographis paniculata* Nees has been traditionally used for centuries in Asia to treat various diseases such as respiratory infection, fever, diarrhea, and bacterial dysentery (Panraksa et al., 2017). Andrographolide (ADO) is a bicyclic diterpenoid lactone and is thought to be the major bioactive component of *A. paniculata*, exerting anti-inflammatory, anti-cancer, and antiviral activities (Wintachai et al., 2015; Panraksa et al., 2017). Previous studies have shown that ADO has activity against several viruses including the human immunodeficiency virus (Calabrese et al., 2000), hepatitis B virus (Chen et al., 2014), hepatitis C virus (Lee et al., 2014), dengue virus (Panraksa et al., 2017), chikungunya virus (Wintachai et al., 2015), and herpes simplex virus (Aromdee et al., 2011). In this study, we evaluated the activity of ADO against the EV-D68 virus. Our results reveal the antiviral activity of ADO and shed light on its utility for the treatment of EV-D68 infections.

## Materials and Methods

### Cells and Viruses

Human rhabdomyosarcoma RD cells (ATCC, CCL-136) were cultured in Dulbecco’s modified Eagle medium (DMEM; Hyclone^TM^, cat no: SH30022.01) supplemented with 10% fetal bovine serum (Biological Industries, REF: 04-001-1) and 1% penicillin/streptomycin solution. EV-D68 prototype Fermon (ATCC, VR-1826) and EV-D68 circulating strains from the 2014 United States outbreak, US/MO/14-18947 (ATCC, VR-1823D) and US/KY/14-18953 (ATCC, VR-1825D), were propagated in RD cells. A mixture of cell and supernatant was collected at approximately 5 days post-infection and subjected to multiple cycles of freezing and thawing. Then, the mixture was clarified by low-speed centrifugation and passed through a 0.22-mm filter, and viral particles were pelleted through a 20% sucrose cushion in a SW28 rotor by centrifugation at 28,000 rpm for 90 min. Purified virions were stored at -80°C until use.

### Cell Viability Assay

RD cells were cultured in 96-well plates with complete medium until they reached 90% confluence. Then, cell culture medium was removed and replaced with complete medium containing 0.4% dimethyl sulfoxide (DMSO) or various concentrations of ADO (MCE, HY-N0191) (0, 1.25, 2.5, 5, 10, 20, and 40 μM). Negative control (only media) and positive control wells (0.4% DMSO) were included on each plate. The cells were incubated for 24 h and then 30 μl MTS was added to each well. Three hours later, the color intensity of each well was detected at 490 nm using a 550 Bio-Rad plate reader. Each data point represents the average of three replicates in cell culture.

### Viral Attachment Assays

For virus attachment experiments, the cells were first washed with cold DMEM, and then EV-D68 viruses were added to the cells. After incubation at 4°C for 2 h, treated cells were washed with cold DMEM to remove unbound viruses. Total RNAs were extracted using an RNeasy Mini Kit (Qiagen). The bound virus RNA was determined by using qRT-PCR.

### Virus Titer Assay

Virus titers were determined by endpoint dilution assay (EPDA). Briefly, RD cells were cultured under standard conditions in 96-well plates at a density of 10,000 cells per well. EV-D68 was serially diluted (10-fold) with DMEM containing 1% FBS and added to cells. Virus titers were determined by the appearance of cytopathic effects (CPEs) in RD cells using a microtitration analysis in accordance with the Reed-Muench method (Reed and Muench, 1938).

### RNA Quantitation by qRT-PCR

Total RNA from cells was isolated using Trizol (Life Technologies) according to the manufacturer’s instructions, including the DNase I digestion step. Samples were incubated in 10 μl of diethyl pyrocarbonate (DEPC)-treated water with 1x RQ1 RNase-Free DNase buffer, l μl of RQ1 RNase-free DNase (Promega), and 4 U of RNase inhibitor (New England Biolabs) for 30 min at 37°C. The DNase activity was inactivated by the addition of 1 μl RQ1 DNase stop solution and incubation at 65°C for 10 min. The RNA was reverse-transcribed by using random primers and Multiscribe reverse transcriptase from the High-Capacity cDNA Archive Kit (Applied Biosystems, Carlsbad, CA, United States), according to the manufacturer’s instructions. The cDNA was either used undiluted or serially diluted in DEPC-treated water before the real-time PCR reaction to ensure that the amplification was within the linear range of detection. The StepOne Real-Time PCR system (Applied Biosystems) was used for qRT-PCR amplification. The reactions were performed under the following conditions: 50°C for 2 min and 95°C for 10 min, followed by 40 cycles of 95°C for 15 s and 60°C for 1 min, followed by a dissociation protocol. Single peaks in the melting curve analysis indicated specific amplicons. qRT-PCR was performed with the following primer sequence: GAPDH forward primer 5′-GCAAATTCCATGGCACCGT-3′; GAPDH reverse primer 5′-TCGCCCCACTTGATTTTGG-3′; EV-D68 forward primer 5′-TGTTCCCACGGTTGAAAACAA-3′; and -EV-D68 reverse primer 5′-TGTCTAGCGTCTCATGGTTTTCAC-3′. IFN-β: 5′-TTGTGCTTCTCCACTACAGC-3′ (forward) and 5′-CTGTAAGTCTGTTAATGAAG-3′ (reverse); ISG56: 5′-CAACCAAGCAAATGTGAGGA-3′ (forward) and 5′-AGGGGAAGCAAAGAAAATGG-3′ (reverse). The relative levels of EV-D68 RNA in different samples were determined using a comparative 2^−ΔΔCT^ method with normalization against the GAPDH gene (Wei et al., 2016).

### Immunoblotting

Cells were harvested at various time points post-infection, washed twice with cold phosphate-buffered saline (PBS), and lysed in lysis buffer (150 mM Tris [pH 7.5] containing 150 mM NaCl, 1% Triton X-100, and complete protease inhibitor cocktail [Roche]) and loading buffer (0.08 M Tris [pH 6.8] containing 2.0% SDS,10% glycerol, 0.1 M DTT, and 0.2% bromphenol blue). The solutions were boiled and vortexed for 10 min and then centrifuged at 14000 *× g* for 10 min. Supernatant proteins were separated by SDS-PAGE and transferred to nitrocellulose membranes using a semidry apparatus (Bio-Rad). The membranes were probed with primary antibodies (anti-EV-D68 VP1 polyclonal antibody [Genetex, GTX132312] and anti-β-actin monoclonal antibody [Sigma, A3853]) at 4°C overnight, followed by incubation with secondary antibodies (alkaline phosphatase-conjugated goat anti-rabbit IgG [Jackson ImmunoResearch Laboratories, code:115-005-045] and goat anti-mouse IgG [Jackson ImmunoResearch Laboratories, code: 115-055-062]) for 1 h at 25°C. The membranes were stained with 5-bromo-4-chloro-3-indolyl phosphate and nitrotetrazolium blue chloride (Sigma-Aldrich) and visualized for band quantification.

### Immunofluorescence and Confocal Microscopy

Cells were grown on glass plates (Nest, cat no.: 801001) in the presence of 10 μM ADO or 0.1% DMSO vehicle overnight until they reached subconfluence. EV-D68 was bound to cells for 45 min at 4°C in DMEM containing 1% FBS. Then, the cells were washed with PBS and incubated at 37°C in 1% DMEM. At various time points, the cells were fixed with 4% paraformaldehyde (PFA) for 15 min, permeabilized with 0.3% Triton X-100 for 5 min, blocked with goat serum (ZSGB-BIO, ZLI-9022) for 1 h 25°C, and finally stained with EV-D68 VP1 antibody (1:250) at 4°C overnight. The next day, the cells were washed five times with PBS and then incubated with Alexa Fluro^®^ 488 conjugated goat anti-rabbit IgG antibody (Life Technologies, A11088) for 1 h at room temperature. DAPI stain (1:100 dilution, Biotopped, top0221) was added 10 min prior to visualization under fluorescence microscopy (Olympus, IX5 or Olympus Fluo-View-1000 with a 100X objective lens) with Fluo View software v. 1.7. Representative cells were selected and photographed.

### Intraendosomal pH Determination

We used amine-reactive pHrodo dyes (Life technologies, cat no.: P35368) to detect changes in the pH of virus-containing endosomes. EV-D68 fermon was purified and dissolved in PBS and incubated with amine-reactive pHrodo dyes for 40 min at room temperature in the dark before infection. RD cells were then seeded on glass plates in the presence of 10 μM ADO or 0.1% DMSO vehicle overnight. Then, the cells were infected with dye-conjugated EV-D68 virus at 4°C for 30 min, washed with PBS and incubated in 37°C, and observed at different time points (1, 2, and 3 h) with a confocal microscope.

### Luciferase Assay

To evaluate the effect of ADO on the viral transcriptional activity of EV-D68, we constructed a promoter-driven firefly luciferase plasmid pT7-5′UTR-pGL3 and expression plasmid p3D-VR_1012_. p5′UTR-Luc (200 ng) was transfected or co-transfected with pEV-D68-3D (800 ng) into cells pre-treated with ADO or 0.1% DMSO vehicle. Luciferase activity was assessed 24 h after transfection. Briefly, the cells were harvested and lysed with passive lysis buffer and then centrifuged at 12000 × *g* for 10 min. Supernatants and luciferase substrate (Promega, E190) were mixed in a 96-well plate and fluorescence was quantified with a Fluoroskan Ascent^TM^ FL instrument (Thermo Fisher, 5210450).

### Statistical Analysis

All data are shown as the mean value and standard deviation of biological replicates (details are explained in figure legends). Statistically significant differences between groups were estimated using two-tailed unpaired Student’s *t-*test with GraphPad Prism (version 6.0) software. *P*-value < 0.05 was considered statistically significant.

## Results

### ADO Inhibits EV-D68 Replication

**FIGURE 1:**
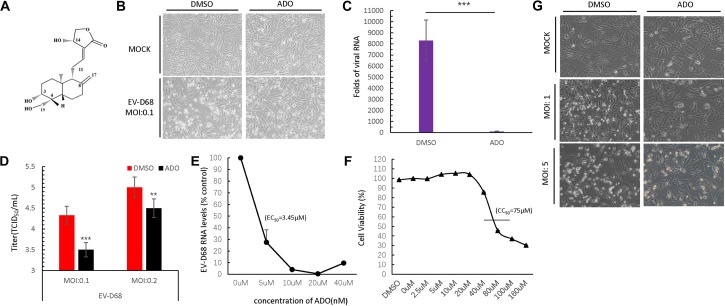
Andrographolide inhibits EV-D68 replication. **(A)** Structure of andrographolide (ADO; molecular formula: C_20_H_30_O_5_). **(B)** RD cells were pre-treated with ADO or DMSO vehicle before infection or mock-infection with EV-D68 virus. The cells were incubated under standard conditions. Cytopathic effects (CPEs) were observed 48 h post-infection. **(C)** qRT-PCR assessment of EV-D68 RNA replication. **(D)** Determination of progeny viron production. Supernatants were collected 48 h post-infection and viral titers were determined by standard plaque assay. **(E)** Effect of ADO on EV-D68 RNA replication (EC_50_ = ∼3.45 μM). **(F)** Cell viability assay. Cellular toxicity was evaluated by MTS assay and is expressed as the percentage relative to DMSO vehicle-treated control cells. Experiments were performed in triplicate. **(G)** RD cells were pretreated the same way as cells in **(B)**, then the cells were infected or mock-infected with EV-D68 virus at an MOI of 1 and 5. CPEs were observed 24 h post-infection. Error bars indicate the standard deviation (^∗^*P* < 0.05; ^∗∗^*P* < 0.01; ^∗∗∗^*P* < 0.001).

Andrographolide is a labdane diterpenoid with broad-spectrum antiviral properties (**Figure 1A**). To investigate the anti-EV-D68 activity of ADO, permissive RD cells were pretreated with 10 μM ADO or 0.1% DMSO vehicle for 16 h, followed by challenge with an EV-D68 prototype Fermon virus (MOI of 0.1). CPEs were detected at 48 h post-infection in vehicle-treated cells; in contrast, ADO-treated cells showed obvious resistance to CPEs (**Figure 1B**). qRT-PCR data indicated that ADO treatment decreased viral RNA replication by 98-fold at 48 h post-infection (**Figure 1C**). Additionally, ADO treatment significantly decreased titers of EV-D68 progeny virus compared to vehicle treatment (**Figure 1D**), suggesting that ADO inhibits the proliferation of EV-D68. In addition, we confirmed that ADO exhibited powerful inhibitory effect on EV-D68 at higher MOI of 1 and 5 (**Figure 1G**). The EC_50_ value of ADO against EV-D68 replication was approximately 3.45 μM (**Figure 1E**). We also measured ADO cytotoxicity using the MTS assay and determined that the median lethal concentration of ADO was 75 μM (**Figure 1F**), which was much higher than the EC_50_ of antiviral activity.

### ADO Inhibits EV-D68 RNA Replication and VP1 Synthesis

**FIGURE 2:**
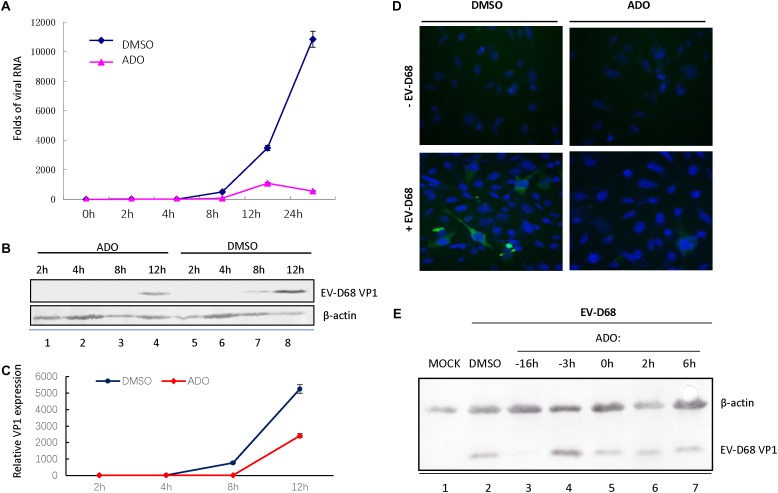
Andrographolide inhibits EV-D68 RNA replication and VP1 synthesis. RD cells were pretreated with andrographolide or DMSO vehicle overnight and subsequently infected with EV-D68. Viral RNA **(A)** and VP1 **(B–D)** were measured at several specific time points. **(A)** Growth curves of EV-D68 in infected cells. **(B,C)** Immunoblotting of VP1 expression at 8 h post-infection. **(D)** Immunofluorescence of VP1 expression at 10 h post-infection. **(E)** RD cells were incubated with 10 μM ADO or vehicle only at the indicated time points before and after infection or mock infection with EV-D68 virus. VP1 was analyzed by western blotting 24 h post-infection, with actin as a control. Experiments were performed in triplicate. Data represent the mean and standard deviation.

We next sought to determine the part of EV-D68 life cycle that was inhibited by ADO treatment. We analyzed EV-D68 growth curves during infection of RD cells (MOI of 10) in the presence of 10 μM ADO or 0.1% DMSO vehicle (**Figure 2A**). Viral RNA was quantified using qRT-PCR at 0, 2, 4, 8, 12, and 24 h post-infection. ADO treatment decreased the amount of intracellular EV-D68 RNA by more than 80% compared to vehicle at 8 h post-infection (**Figure 2A**). At 24 h post-infection, the amount of EV-D68 RNA in vehicle-treated cells was 19-fold higher than that in ADO-treated cells (**Figure 2A**). Consistent with this result, there was an observable reduction in EV-D68 VP1 protein expression in ADO-treated cells compared to vehicle-treated cells on immunoblotting (**Figures 2B,C**) and immunofluorescence assays (**Figure 2D**). To clarify the dynamics of ADO against EV-D68, a time-of-addition analysis was undertaken, with ADO treatment at 16 and 3 h prior to infection and 0, 2, and 6 h post-infection. VP1 expression was evaluated by immunoblotting. The results indicated that ADO treatment post-infection had no obvious effect on EV-D68 (**Figure 2E**). All these data suggested that ADO treatment impeded the early stage of EV-D68 infection.

### ADO Does Not Influence EV-D68 3D-5′ UTR Activity or Innate Immune Responses

**FIGURE 3:**
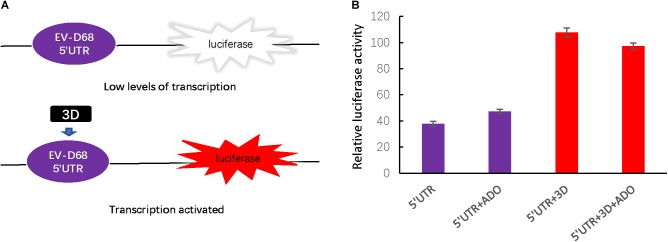
Andrographolide does not influence EV-D68 3D-dependent 5′ UTR transcriptional activity or the cellular innate immune response. **(A)** To assess the effect of andrographolide on EV-D68 transcription, we generated a firefly luciferase reporter assay of 5′ UTR activity. **(B)** Assay of 3D-dependent 5′ UTR activation. p5′UTR-luc (200 ng) was transfected or co-transfected with pEV-D68-3D (800 ng) into HEK293T cells pretreated with ADO or DMSO vehicle in a 12-well plate. Luciferase activity was detected 24 h post-transfection.

The 5′ UTR region of enteroviruses is a determinant for viral translation and RNA replication, and the activity of this region can be enhanced by viral polymerase 3D protein. To assess viral 3D protein-dependent 5′ UTR activation, we next established a firefly luciferase reporter assay (**Figure 3A**) and found that ADO treatment had no influence on 5′ UTR activity regardless of 3D expression (**Figure 3B**).

**FIGURE 4:**
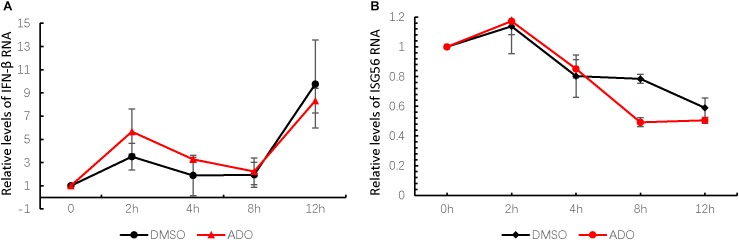
Andrographolide does not alter EV-D68-induced innate immune responses at the cellular level. Gene expression of IFN-β **(A)** and ISG-56 **(B)**, respectively, as indicators of virus-stimulated innate immune response. mRNA was quantified by qRT-PCR at 0, 2, 4, 8, and 12 h post-infection. Error bars indicate the standard deviation.

Previous studies have characterized ADO as a strong plant-derived immunomodulator that affects the NF-κB and JAK-STAT signaling pathways, which are involved in host innate immune responses (Ding et al., 2017). Therefore, we next measured endogenous mRNA levels of interferon-β (IFN-β) and an interferon-stimulated gene ISG56 at 0, 2, 4, 8, and 12 h post-infection (**Figures 4A,B**). IFN-β mRNA was increased at 12 h post-infection, suggesting that EV-D68 infection produced innate immune activation in permissive cells (**Figure 4A**). ADO treatment at a concentration of 10 μM did not have a significant effect on IFN-β or ISG56 mRNA expression during EV-D68 infection in RD cells (**Figures 4A,B**), indicating that ADO did not alter EV-D68-induced innate immune responses at the cellular level.

### ADO Does Not Influence EV-D68 Entry Into Cells

**FIGURE 5:**
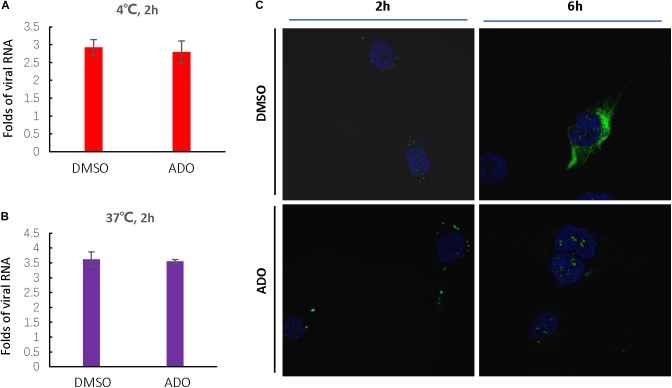
Andrographolide inhibits EV-D68 replication in a post-entry step. Andrographolide had no effect on EV-D68 attachment or entry into cells. Cells were pre-treated with ADO or DMSO vehicle in 12-well plates for 16 h under standard conditions. **(A)** Cells were incubated at 4°C for 30 min before infection and then incubated at 4°C for 2 h to facilitate virus attachment. **(B)** RD cells were infected with EV-D68 and incubated at 37°C for 2 h. qRT-PCR was performed to quantify viral RNA. **(C)** Assay of intracellular VP1 accumulation. Cells were fixed at 2 and 6 h post-infection, and VP1 was observed by confocal microscopy. Experiments were performed in triplicate. Error bars indicate the standard deviation.

Based on the above studies, we determined that ADO likely inhibited a step in the EV-D68 lifecycle occurring prior to viral RNA replication and protein expression. Therefore, we next investigated whether ADO inhibited EV-D68 replication at the entry step. Viral attachment and entry experiments were performed in the presence of 10 μM ADO or 0.1% DMSO vehicle. Virus attachment and entry were assessed by quantification of intracellular EV-D68 RNA at 2 h post-infection. Repeated experiments demonstrated that pre-treatment of cells with ADO had no detectable effect on viral attachment (**Figure 5A**) or entry (**Figure 5B**). Next, we detected intracellular VP1 protein expression using immunofluorescence at 2 and 6 h post-infection. Consistent with previous result, there was no effect of ADO treatment at 2 h post-infection; however, ADO treatment significantly decreased EV-D68 VP1 protein expression at 6 h post-infection (**Figure 5C**). These results collectively demonstrated that ADO inhibited EV-D68 RNA replication and protein accumulation in a post-entry step.

### ADO Inhibits the Acidification of EV-D68-Containing Endosomes

**FIGURE 6:**
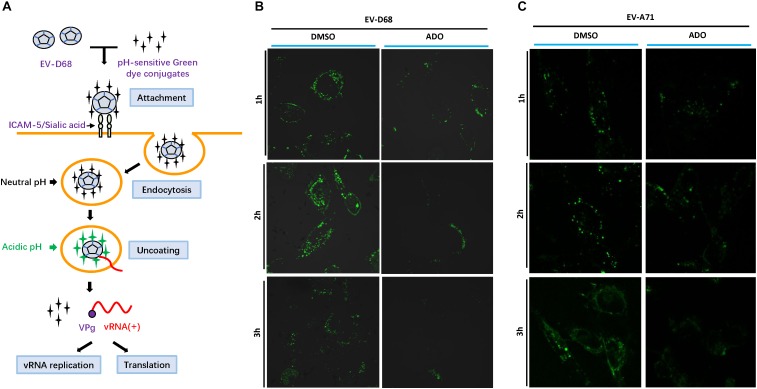
Andrographolide interferes with endosome maturation. **(A)** Viruses are endocytosed at the cell surface before trafficking toward the nucleus. Gradual acidification occurs alongside endosome maturation. Viral proteins require low pH to undergo conformational changes or protease activation that permit disruption of the endosome membrane and the release of viral particles into the cytoplasm. **(B,C)** Assay of endosome acidification. EV-D68 and EV-A71 were incubated with pH-sensitive green dye and then infected into pre-treated RD cells. Fluorescence was detected in live cells using Alexa Fluor 488 filters and confocal microscopy at specific time points (1, 2, and 3 h post-infection). Fluorescence increased with acidity of the surrounding environment.

Enteroviruses share a common mechanism in the early steps of viral infection that involves specific receptor binding, viral entry through an endocytic pathway, and subsequent travel through the cytoplasm encased in an endosomal compartment. In most cases, endosomes that contain pathogens undergo maturation to lysosomes to degrade the pathogens. Therefore, the virus must exit the endosome to escape degradation. Viruses use the low pH of endosomes to activate penetration proteins or undergo conformational changes that facilitate escape (Lagache et al., 2012). To track the process of EV-D68 endocytosis in the presence or absence of ADO treatment, we established a pH-sensitive reporter system during EV-D68 infection by using an acidic pH-sensitive green dye. The dye has a pH-sensitive fluorescence emission that increases in intensity with increasing acidity, with peak fluorescence in the range pH 5–8 (i.e., the known range of endosome vesicle acidification; **Figure 6A**). Fluorescence intensity peaked at around 2 h post-infection and was quickly quenched by 3 h post-infection in vehicle-treated RD cells (**Figure 6B**). In contrast, ADO-treated cells exhibited limited fluorescence, suggesting that ADO inhibited the maturation process of EV-D68 endocytosis. Furthermore, we demonstrated that ADO treatment can inhibit the acidification of virus-containing endosome of an enteric enterovirus EV-A71 (**Figure 6C**). Thus, applying a broadly antiviral activity of ADO against diverse viruses via inhibiting endosome acidification.

### ADO Inhibits Isolated Circulating Strains of EV-D68

**FIGURE 7:**
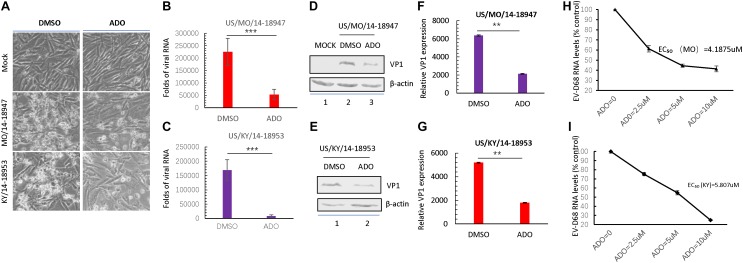
Andrographolide inhibits isolated circulating strains of EV-D68. **(A)** RD cells were pre-treated with 10 μM andrographolide or DMSO vehicle and then infected with US/MO/14-18947 or US/KY/14-18953. Cells were incubated under standard conditions. Cytopathic effects (CPEs) were observed 48 h post-infection. **(B,C)** qRT-PCR analysis of viral RNA. **(D–G)** Immunoblotting of VP1 expression at 24 h post-infection with the US/MO/14-18947 **(D,F)** and US/KY/14-18953 strains **(E,G)**. **(H,I)** Effect of ADO on RNA replication of EV-D68 circulating strains. US/MO/14-18947:EC_50_ = ∼4.1875 μM **(H)**; US/KY/14-18953: EC_50_ = ∼5.807 μM. The experiment was performed three times. Error bars indicate the standard deviation (^∗^*P* < 0.05; ^∗∗^*P* < 0.01; ^∗∗∗^*P* < 0.001).

Recent studies presented by our lab and others have demonstrated that the EV-D68 prototype virus and circulating strains have different sensitivities for sialic acid-mediated viral replication. Therefore, we examined the antiviral activity of ADO against viral infection by primary EV-D68 isolates (US/MO/14-18947 [MO] and US/KY/14-18953 [KY]). The cells were harvested at 24 h post-infection for immunoblotting of EV-D68 VP1, and CPEs were observed 48 h post-infection. The results indicated that ADO protected RD cells against both circulating strains of EV-D68 as evidenced by decreases in related CPEs (**Figure 7A**). Further, ADO treatment significantly decreased intracellular amounts of viral RNA after infection with the MO (**Figure 7B**) and KY strains (**Figure 7C**) compared to vehicle treatment. Finally, ADO treatment significantly decreased VP1 protein expression after infection with the MO (**Figures 7D,F**) and KY strains (**Figures 7E,G**) compared to vehicle treatment. The EC_50_ values of ADO against MO and KY replication were approximately 4.19 μM (**Figure 7H**) and 5.807 μM (**Figure 7I**), which was close to that of AOD against EV-D68 prototype Fermon. In summary, ADO exerted broad antiviral effects against both prototype and circulating EV-D68 viruses.

## Discussion

To our knowledge, this is the first study to report the inhibition of viral endocytosis by ADO, a major bioactive constituent of *A. paniculata*. ADO treatment conferred significant antiviral activity against EV-D68 virus without producing significant toxicity in RD cells. ADO treatment was associated with significant decreases in viral RNA replication and protein synthesis. Notably, ADO had no influence on viral attachment, viral endocytosis processes, or innate immune activation in infected cells. By using a pH-sensitive fluorescent dye conjugate to track virus endocytosis in live cells, we revealed that ADO interfered with viral cytoplasmic traffic by preventing vesicle acidification.

*Andrographis paniculata* has long been an important constituent of traditional herbal medicine in Asia (Kumar et al., 2004) and Scandinavia (Jayakumar et al., 2013), with several studies demonstrating the antiviral, antimicrobial, anti-parasitic, and anti-inflammatory effects of ADO as a principal active component. Research has demonstrated the therapeutic utility of ADO in respiratory infection by influenza A virus (IAV) by inhibition of IAV-induced RIG-I like receptor signaling pathway in human bronchial epithelial cells (Yu et al., 2014). However, the mechanisms by which ADO exerts broad-spectrum antiviral activity have been poorly defined in previous studies. In our experiments, ADO treatment did not influence EV-D68 infection-mediated innate immune activation as represented by the induction of IFN-β and ISG56.

In this study, we analyzed the growth curves of EV-D68 RNA replication and VP1 synthesis during infection and found that ADO dramatically decreased intracellular viral RNA copy numbers and VP1 expression. This finding was consistent with previous studies demonstrating the ability of ADO to prevent the infection of diverse viruses by inhibiting viral protein expression (HSV-1, HCV, HPV, and CHIKV) and viral genome replication (HBV, HSV-1, and CHIKV). The 5′ UTR activity of enteroviruses including poliovirus, EV71, CV-A16, and rhinovirus is crucial for viral RNA replication and protein translation (Gamarnik and Andino, 2000). In this study, we found that ADO did not affect the 5′ UTR activity of EV-D68 in a viral RNA polymerase 3D-dependent 5′ UTR reporter assay, indicating that ADO targeted a step prior to the initiation of viral RNA and protein syntheses.

A previous study of the antiviral activity of ADO and its analogs showed that ADO prevented the entry of HSV-1 into cells (Aromdee et al., 2011). The life cycle of an enterovirus starts with binding of a cell surface receptor. Previous studies have demonstrated that sialic acid is required for the cellular entry of certain EV-D68 viruses (Liu et al., 2015a). To this end, our lab identified ICAM-5 as a functional receptor for both sialic acid-sensitive and insensitive EV-D68 strains (Wei et al., 2016). In this study, we found that ADO did not influence the entry of EV-D68 into RD cells.

Enterovirus binds its specific receptor on the cell surface and then enters the host cell through receptor-mediated endocytosis. Subsequently, receptor binding or changes in pH in the virus-containing endosome trigger virus uncoating. Notably, EV-D68 is a unique respiratory enterovirus that is sensitive to acidic conditions and prefers lower culture temperatures (Oberste et al., 2004). Consistent with these properties, we found that acidification of the virus-containing endocytic vesicle was a critical step inhibited by ADO that ultimately inhibited EV-D68 infection. Consistent with this observation, previous studies have indicated that drugs inhibiting endosome acidification (Bafilomycin A1 and concanamycin A) prevented EV71 multiplication. Therefore, disrupting endosome acidification may represent an important antiviral strategy for therapeutic development against EV-D68. Furthermore, ADO has been shown to inhibit autophagosome–lysosome fusion and suspended autophagy processes (Zhou et al., 2012). Future studies are needed to identify the cellular targets of ADO that inhibit endosome/autophagosome maturation.

The work described herein provides compelling evidence that ADO inhibits EV-D68 replication by preventing endosome acidification as a novel antiviral property. Our results show virus–host interactions and highlight the potential therapeutic utility of ADO as a clinical therapeutic against EV-D68 infection.

## Author Contributions

DyW, HG, JC, and DW performed the experiments. DyW, HG, PG, and WW analyzed the data. WW, DyW, PG, and BL wrote the paper with help from all authors. WW, PG, and BL directed the project.

## Conflict of Interest Statement

The authors declare that the research was conducted in the absence of any commercial or financial relationships that could be construed as a potential conflict of interest.
